# 131-Radioiodine Therapy for Graves Disease in a Patient With Down Syndrome and Non-operable Congenital Heart Disease

**DOI:** 10.7759/cureus.80396

**Published:** 2025-03-11

**Authors:** Ketki Ambulkar, Vyankatesh Shivane, Rohit Barnabas, Vikram Lele, Tushar Bandgar

**Affiliations:** 1 Endocrinology, Diabetes, and Metabolism, King Edward Memorial Hospital and Seth Gordhandas Sunderdas Medical College, Mumbai, IND; 2 Diabetology and Endocrinology, Sadhana Diabesity Clinic, Mumbai, IND; 3 Endocrinology, King Edward Memorial Hospital and Seth Gordhandas Sunderdas Medical College, Mumbai, IND; 4 Nuclear Medicine, Jaslok Hospital, Mumbai, IND

**Keywords:** congenital heart disease, down syndrome, graves disease, iodine-131, radioiodine therapy

## Abstract

Thyroid dysfunctions are prevalent in Down syndrome (DS), with hypothyroidism being the most frequent occurrence, whereas hyperthyroidism is rare. DS patients face an increased risk of comorbidities, including gastrointestinal, cardiac, and pulmonary anomalies, as well as developmental delays, endocrine abnormalities, and malignancies, complicating their treatment and management. The published data on pediatric hyperthyroidism in DS from India comprise five case reports with six patients. We report a case of a 13-year-old male child with DS with Graves disease (GD), who had inoperable congenital heart disease (CHD) and was administered iodine-131 radioiodine therapy as a definitive treatment for GD.

## Introduction

Down syndrome (DS), first described by John Langdon Down in 1866, is the most common chromosomal anomaly caused by the presence of a third copy of chromosome 21 [1]. It affects approximately one in 850-1000 live births in India and is associated with intellectual disability and congenital abnormalities [2]. Advanced maternal age (≥ 35 years old) is a known risk factor for DS [1]. DS patients face an increased risk of comorbidities, including gastrointestinal, cardiac, and pulmonary anomalies, as well as developmental delays, endocrine abnormalities, and malignancies [3]. Endocrine disorders, particularly thyroid dysfunctions such as hypothyroidism and Graves disease (GD), are prevalent, with hypothyroidism being more common. Thyroid abnormalities are found in 4-8% of children with DS. Hyperthyroidism can lead to complications such as osteoporosis, psychomotor abnormalities, and heart disease if untreated.

A high prevalence of congenital heart disease (CHD) is observed in children with DS. Studies indicate that approximately 41.8% to 72.73% of DS children have CHD, with ventricular septal defect (VSD) and patent ductus arteriosus (PDA) being common types [4]. The presence of CHD significantly affects the quality of life and mortality in DS patients. Socioeconomic status (SES), especially education, income, employment, and environment, may have a negative effect on the diagnosis and management of cardiovascular (CV) conditions. Lower SES can have increased CV risk similar to traditional risk factors; this may be seen in patients with CHD in DS. However, further studies are required in this area [5]. We present the case of a DS pediatric patient with CHD with GD.

## Case presentation

**Figure 1:**
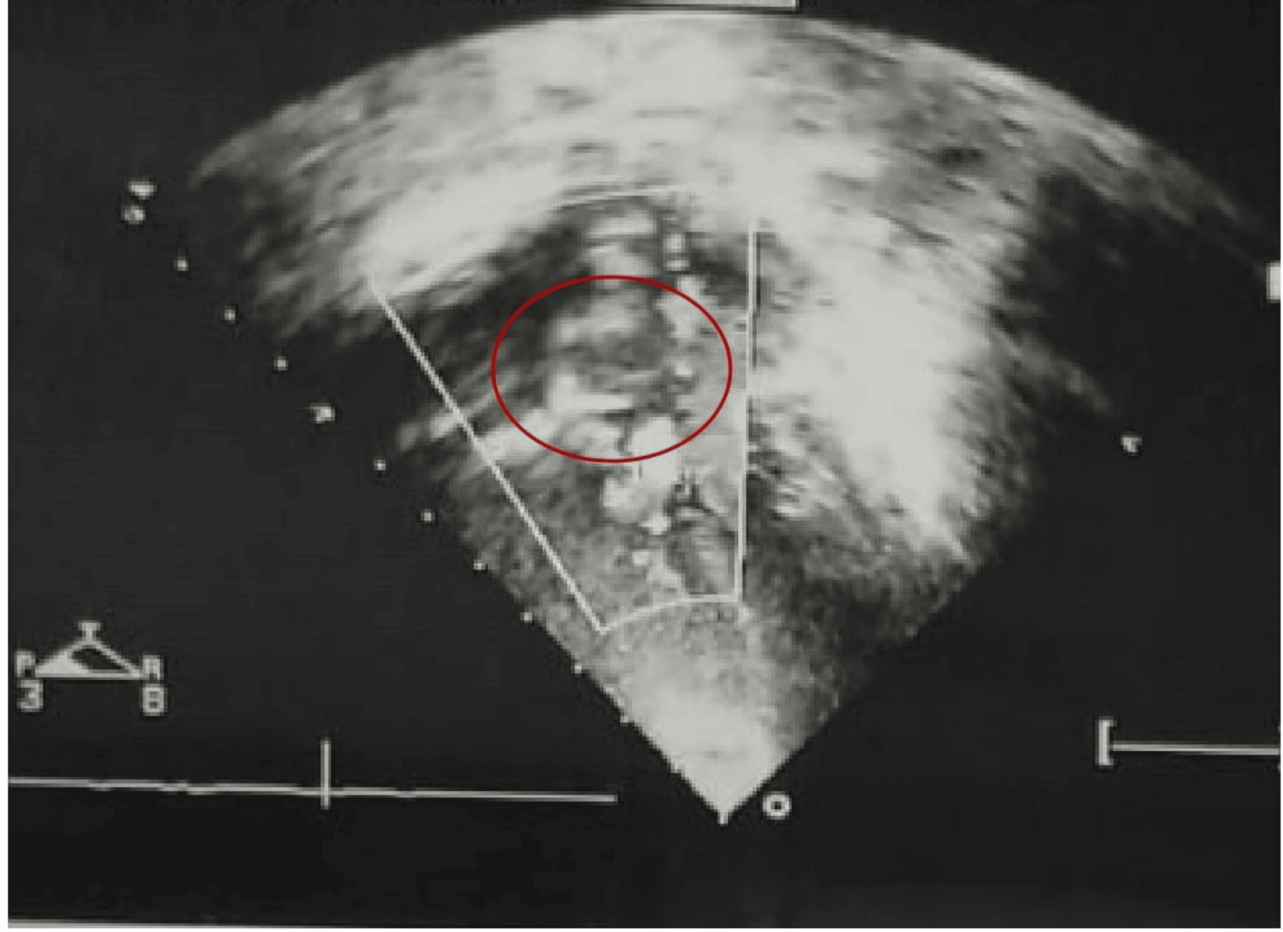
Echocardiography of the patient showing an 8-mm nonrestrictive ventricular septal defect with severe pulmonary hypertension and Eisenmenger syndrome

A 13-year-old boy with DS presented with weight loss (approximately 5 kg in six months), dyspnea (New York Heart Association (NYHA) class II), and palpitations. He had an 8 mm nonrestrictive subaortic VSD with severe pulmonary hypertension and Eisenmenger syndrome, not amenable to surgical treatment (Figure 1). He was under regular cardiology follow-up. Laboratory results indicated hyperthyroidism, prompting a referral to endocrinology. Clinical history was insignificant except for the mother's age at conception, which was 37 years. Physical examination revealed moist hands, fine tremors, sinus tachycardia, goiter, and pansystolic murmur. His weight was 30 kg, height 134 cm, and pulse rate 110 bpm. Thyroid function tests confirmed hyperthyroidism, with a free T4 of 26 pmol/L and TSH (thyroid-stimulating hormone) of 0.05 µIU/mL. Thyroid autoantibodies (TRAb, AMA, and ATG) were positive, leading to a diagnosis of GD.

Initially, carbimazole (CMZ) 20 mg daily was prescribed, later increased to 30 mg per day. Despite six months of treatment, the patient remained hyperthyroid, so radioactive iodine therapy (RAI) was administered (10 mCi of 131-I). Consent was obtained from his mother. Post-therapy, CMZ was continued on a tapering dose for three months, after which it was stopped. Following treatment, he gained 14 kg, and his pulse normalized. Eight months later, he developed hypothyroidism and was started on thyroxine replacement therapy. His dyspnea improved, and a recent echocardiography showed no new cardiac changes. He is now on regular follow-up for hypothyroid management (Table 1).

**Table 1:** Laboratory values and treatment timeline of the case FT3: free T3; FT4: free T4: LT4: levothyroxine; NMZ: neomercazole; RAI: radioiodine therapy; TSH: thyroid-stimulating hormone

Parameter (Reference Range)	0 months	2 months	4 months	6 months	8 months	10 months
TSH (0.51-4.3 µIU/mL)	<0.005	<0.005	0.021	66.1	98.04	70.02
FT3 (2.56-5.01 pg/mL)	5.14	5.12	3.93	0.7	<0.3	2.14
FT4 (0.98-1.63 ng/dL)	1.29	1.39	0.90	0.17	0.08	0.556
Treatment	NMZ 20 mg	NMZ 30 mg	NMZ 25 mg	NMZ 30 mg RAI 10 mCi	LT4 37.5 µg	LT4 100 µg

## Discussion

While DS patients are predisposed to autoimmune diseases, hyperthyroidism is rare in this population [1]. Approximately 60 pediatric cases of DS with hyperthyroidism are documented, with five case reports from India [6-9]. Studies in India show a GD prevalence of 1-10% in DS patients, significantly higher than in the general population [6]. GD in DS patients tends to present in pediatric age compared to the general population (6.5% vs. 1.07%), with no gender predominance and a milder clinical course. This may be attributed to increased awareness among physicians and frequent monitoring [10].

Pharmacotherapy is the initial treatment in children and adolescents with GD, but remission is only seen in 20-30% of cases after two years of continuous treatment [11]. In our case, it required six months of high-dose CMZ (30 mg) for biochemical normalization of free T4. Surgery is generally limited in DS patients due to anesthesia risks associated with craniofacial anomalies and short necks. It is only considered in cases with serious antithyroid drug side effects or when rapid control of thyrotoxicosis is necessary.

In complex cases like ours, where surgery posed risks due to cardiac comorbidity, RAI was a suitable definitive treatment. A Spanish study on DS reported 12 hyperthyroid cases (6.5 per 1000 patients), all managed with radioiodine therapy after unsuccessful pharmacotherapy [12]. In a study by Damle et al., a patient with patent ductus arteriosus received RAI (5 mCi) as a definitive treatment [9]. RAI is advantageous for DS patients due to its single-dose administration, minimizing compliance issues and reducing the likelihood of relapse. Available in capsule form, RAI is also a convenient outpatient procedure.

## Conclusions

Although hyperthyroidism is rarer in DS than hypothyroidism, it remains more common in DS patients than in the general population. In some cases, antithyroid drugs may fail to achieve remission, necessitating RAI as a definitive treatment. DS patients often present with increased morbidity, making RAI a valuable therapeutic option for achieving control with minimal compliance burden.
